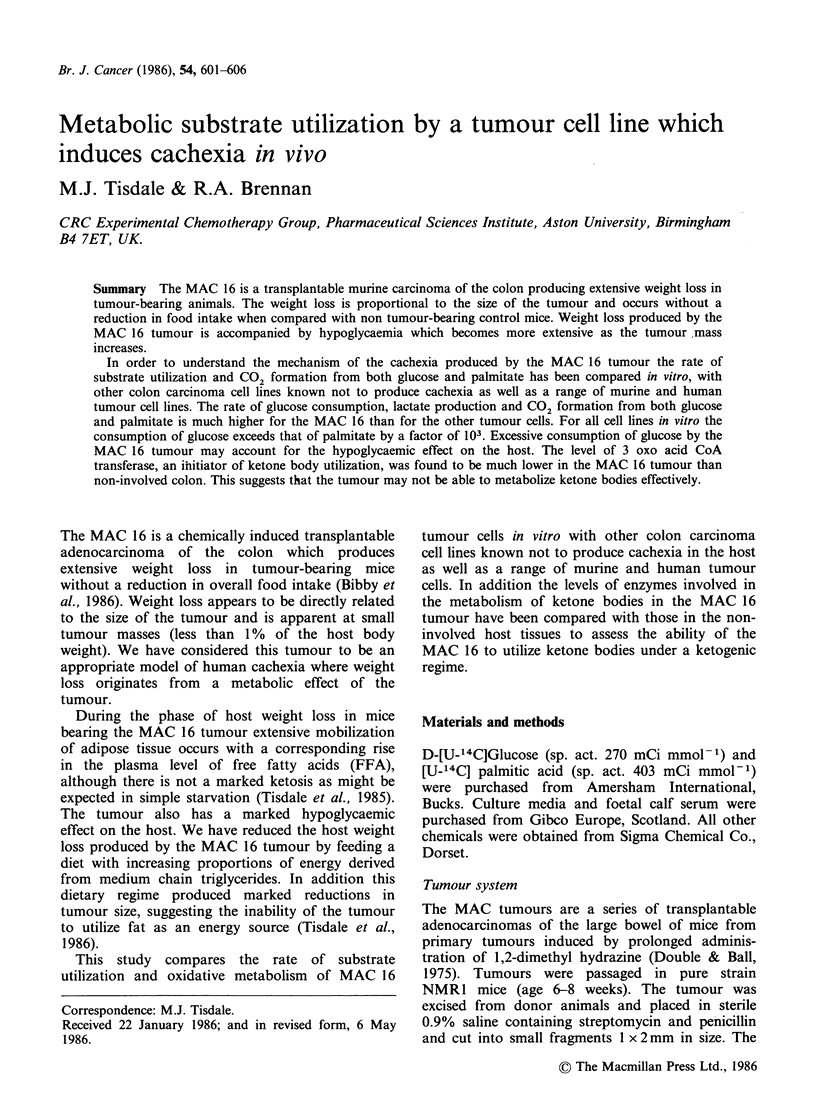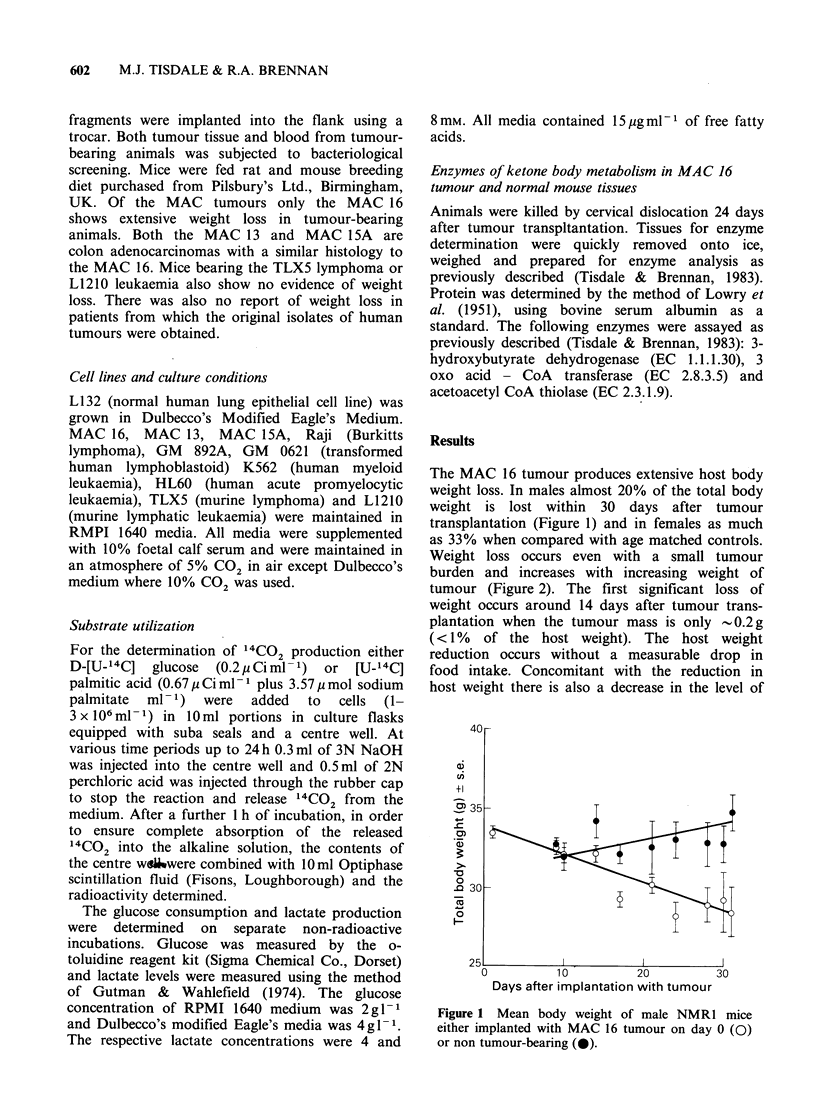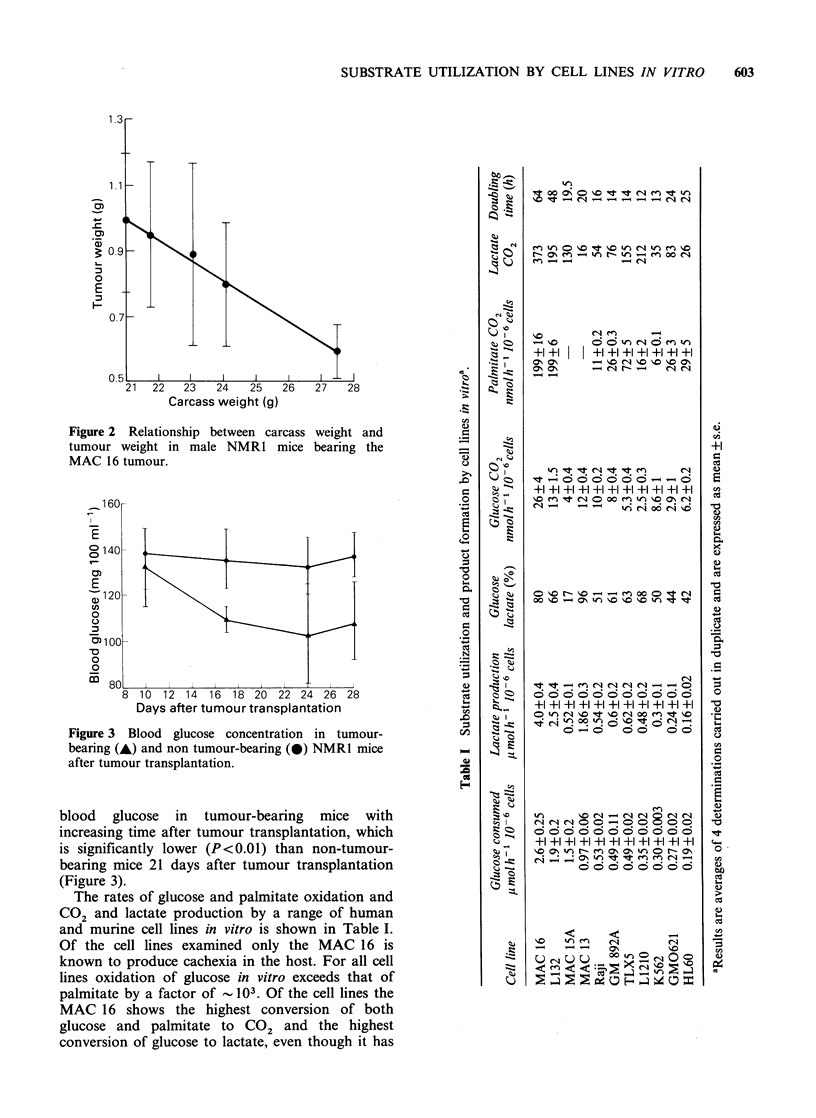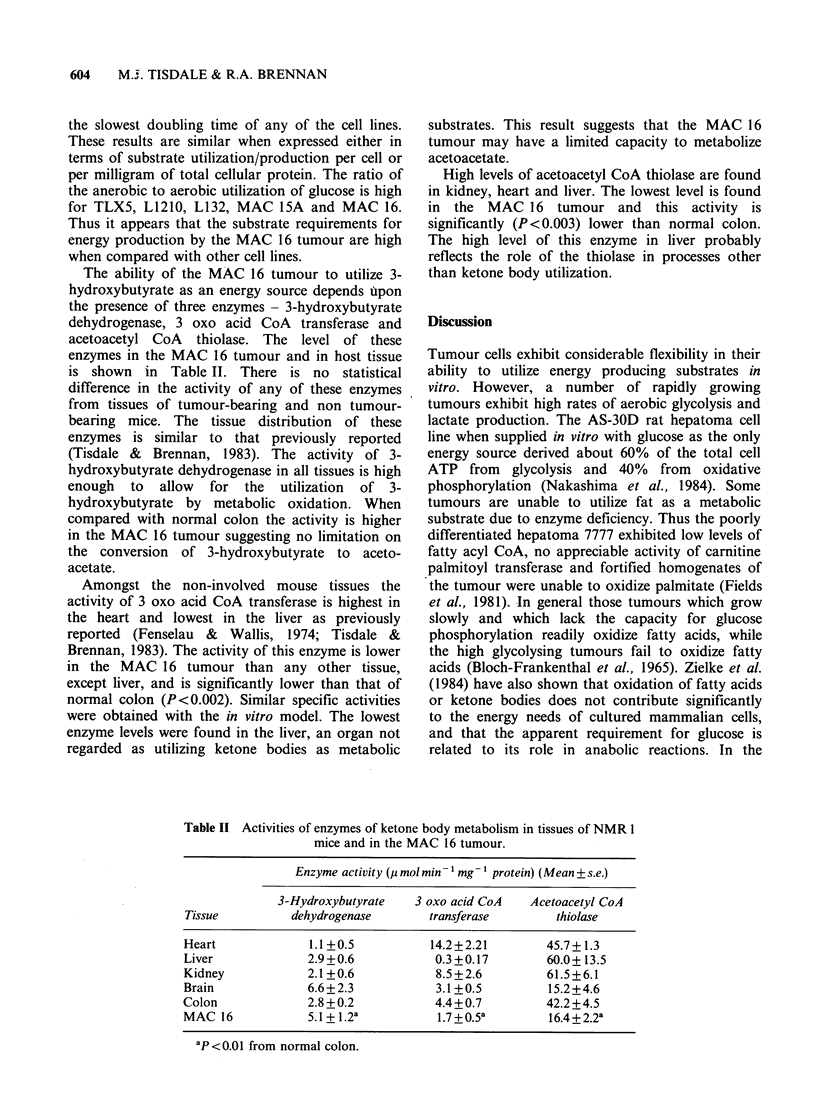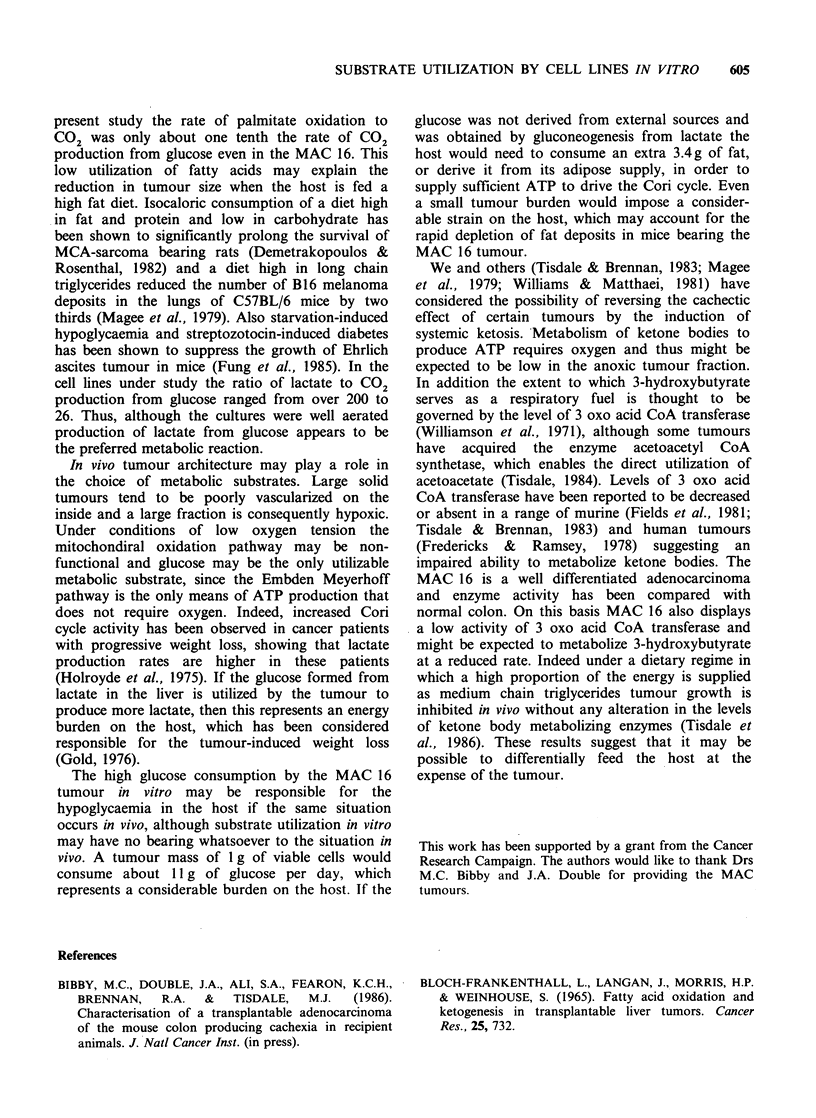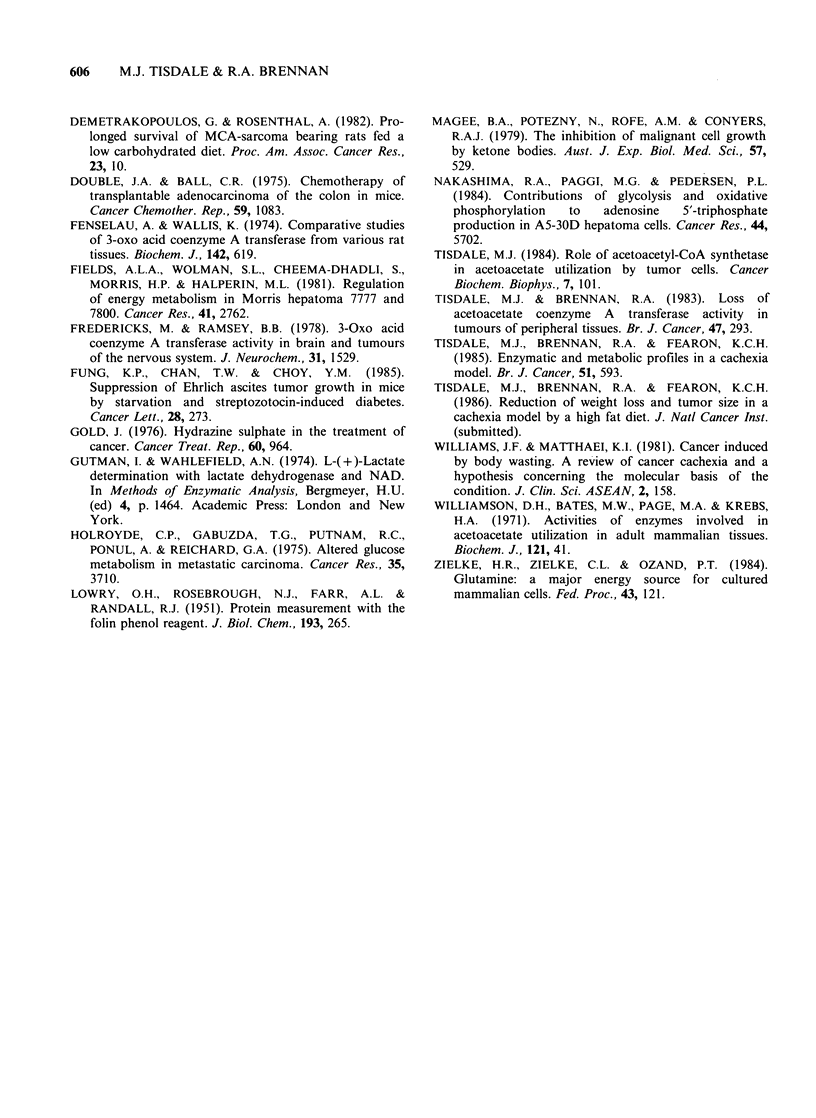# Metabolic substrate utilization by a tumour cell line which induces cachexia in vivo.

**DOI:** 10.1038/bjc.1986.215

**Published:** 1986-10

**Authors:** M. J. Tisdale, R. A. Brennan

## Abstract

The MAC 16 is a transplantable murine carcinoma of the colon producing extensive weight loss in tumour-bearing animals. The weight loss is proportional to the size of the tumour and occurs without a reduction in food intake when compared with non tumour-bearing control mice. Weight loss produced by the MAC 16 tumour is accompanied by hypoglycaemia which becomes more extensive as the tumour mass increases. In order to understand the mechanism of the cachexia produced by the MAC 16 tumour the rate of substrate utilization and CO2 formation from both glucose and palmitate has been compared in vitro, with other colon carcinoma cell lines known not to produce cachexia as well as a range of murine and human tumour cell lines. The rate of glucose consumption, lactate production and CO2 formation from both glucose and palmitate is much higher for the MAC 16 than for the other tumour cells. For all cell lines in vitro the consumption of glucose exceeds that of palmitate by a factor of 10(3). Excessive consumption of glucose by the MAC 16 tumour may account for the hypoglycaemic effect on the host. The level of 3 oxo acid CoA transferase, an initiator of ketone body utilization, was found to be much lower in the MAC 16 tumour than non-involved colon. This suggests that the tumour may not be able to metabolize ketone bodies effectively.


*Br. J. Cancer* (1986), **54**, 601–606

### Metabolic substrate utilization by a tumour cell line which induces cachexia *in vivo*

M.J. Tisdale & R.A. Brennan

*CRC Experimental Chemotherapy Group, Pharmaceutical Sciences Institute, Aston University, Birmingham B4 7ET, UK.*

**Summary** The MAC 16 is a transplantable murine carcinoma of the colon producing extensive weight loss in tumour-bearing animals. The weight loss is proportional to the size of the tumour and occurs without a reduction in food intake when compared with non tumour-bearing control mice. Weight loss produced by the MAC 16 tumour is accompanied by hypoglycaemia which becomes more extensive as the tumour mass increases.

In order to understand the mechanism of the cachexia produced by the MAC 16 tumour the rate of substrate utilization and $CO_2$ formation from both glucose and palmitate has been compared *in vitro*, with other colon carcinoma cell lines known not to produce cachexia as well as a range of murine and human tumour cell lines. The rate of glucose consumption, lactate production and $CO_2$ formation from both glucose and palmitate is much higher for the MAC 16 than for the other tumour cells. For all cell lines *in vitro* the consumption of glucose exceeds that of palmitate by a factor of $10^3$. Excessive consumption of glucose by the MAC 16 tumour may account for the hypoglycaemic effect on the host. The level of 3 oxo acid CoA transferase, an initiator of ketone body utilization, was found to be much lower in the MAC 16 tumour than non-involved colon. This suggests that the tumour may not be able to metabolize ketone bodies effectively.

The MAC 16 is a chemically induced transplantable adenocarcinoma of the colon which produces extensive weight loss in tumour-bearing mice without a reduction in overall food intake (Bibby *et al.*, 1986). Weight loss appears to be directly related to the size of the tumour and is apparent at small tumour masses (less than 1% of the host body weight). We have considered this tumour to be an appropriate model of human cachexia where weight loss originates from a metabolic effect of the tumour.

During the phase of host weight loss in mice bearing the MAC 16 tumour extensive mobilization of adipose tissue occurs with a corresponding rise in the plasma level of free fatty acids (FFA), although there is not a marked ketosis as might be expected in simple starvation (Tisdale *et al.*, 1985). The tumour also has a marked hypoglycaemic effect on the host. We have reduced the host weight loss produced by the MAC 16 tumour by feeding a diet with increasing proportions of energy derived from medium chain triglycerides. In addition this dietary regime produced marked reductions in tumour size, suggesting the inability of the tumour to utilize fat as an energy source (Tisdale *et al.*, 1986).

This study compares the rate of substrate utilization and oxidative metabolism of MAC 16 tumour cells *in vitro* with other colon carcinoma cell lines known not to produce cachexia in the host as well as a range of murine and human tumour cells. In addition the levels of enzymes involved in the metabolism of ketone bodies in the MAC 16 tumour have been compared with those in the non-involved host tissues to assess the ability of the MAC 16 to utilize ketone bodies under a ketogenic regime.

### Materials and methods

D-[U-$^{14}$C]Glucose (sp. act. 270 mCi mmol$^{-1}$) and [U-$^{14}$C] palmitic acid (sp. act. 403 mCi mmol$^{-1}$) were purchased from Amersham International, Bucks. Culture media and foetal calf serum were purchased from Gibco Europe, Scotland. All other chemicals were obtained from Sigma Chemical Co., Dorset.

### *Tumour system*

The MAC tumours are a series of transplantable adenocarcinomas of the large bowel of mice from primary tumours induced by prolonged administration of 1,2-dimethyl hydrazine (Double & Ball, 1975). Tumours were passaged in pure strain NMRI mice (age 6–8 weeks). The tumour was excised from donor animals and placed in sterile 0.9% saline containing streptomycin and penicillin and cut into small fragments 1 × 2 mm in size. The

Correspondence: M.J. Tisdale.
Received 22 January 1986; and in revised form, 6 May 1986.

© The Macmillan Press Ltd., 1986

fragments were implanted into the flank using a trocar. Both tumour tissue and blood from tumour-bearing animals was subjected to bacteriological screening. Mice were fed rat and mouse breeding diet purchased from Pilsbury's Ltd., Birmingham, UK. Of the MAC tumours only the MAC 16 shows extensive weight loss in tumour-bearing animals. Both the MAC 13 and MAC 15A are colon adenocarcinomas with a similar histology to the MAC 16. Mice bearing the TLX5 lymphoma or L1210 leukaemia also show no evidence of weight loss. There was also no report of weight loss in patients from which the original isolates of human tumours were obtained.

*Cell lines and culture conditions*

L132 (normal human lung epithelial cell line) was grown in Dulbecco's Modified Eagle's Medium. MAC 16, MAC 13, MAC 15A, Raji (Burkitts lymphoma), GM 892A, GM 0621 (transformed human lymphoblastoid) K562 (human myeloid leukaemia), HL60 (human acute promyelocytic leukaemia), TLX5 (murine lymphoma) and L1210 (murine lymphatic leukaemia) were maintained in RMPI 1640 media. All media were supplemented with 10% foetal calf serum and were maintained in an atmosphere of 5% $CO_2$ in air except Dulbecco's medium where 10% $CO_2$ was used.

*Substrate utilization*

For the determination of $^{14}CO_2$ production either D-[U-$^{14}$C] glucose (0.2 $\mu$Ci ml$^{-1}$) or [U-$^{14}$C] palmitic acid (0.67 $\mu$Ci ml$^{-1}$ plus 3.57 $\mu$mol sodium palmitate ml$^{-1}$) were added to cells (1–3 $\times$ 10$^6$ ml$^{-1}$) in 10 ml portions in culture flasks equipped with suba seals and a centre well. At various time periods up to 24 h 0.3 ml of 3N NaOH was injected into the centre well and 0.5 ml of 2N perchloric acid was injected through the rubber cap to stop the reaction and release $^{14}CO_2$ from the medium. After a further 1 h of incubation, in order to ensure complete absorption of the released $^{14}CO_2$ into the alkaline solution, the contents of the centre well were combined with 10 ml Optiphase scintillation fluid (Fisons, Loughborough) and the radioactivity determined.

The glucose consumption and lactate production were determined on separate non-radioactive incubations. Glucose was measured by the o-toluidine reagent kit (Sigma Chemical Co., Dorset) and lactate levels were measured using the method of Gutman & Wahlefield (1974). The glucose concentration of RPMI 1640 medium was 2 g l$^{-1}$ and Dulbecco's modified Eagle's media was 4 g l$^{-1}$. The respective lactate concentrations were 4 and 8 mM. All media contained 15 $\mu$g ml$^{-1}$ of free fatty acids.

*Enzymes of ketone body metabolism in MAC 16 tumour and normal mouse tissues*

Animals were killed by cervical dislocation 24 days after tumour transpltantation. Tissues for enzyme determination were quickly removed onto ice, weighed and prepared for enzyme analysis as previously described (Tisdale & Brennan, 1983). Protein was determined by the method of Lowry *et al.* (1951), using bovine serum albumin as a standard. The following enzymes were assayed as previously described (Tisdale & Brennan, 1983): 3-hydroxybutyrate dehydrogenase (EC 1.1.1.30), 3 oxo acid – CoA transferase (EC 2.8.3.5) and acetoacetyl CoA thiolase (EC 2.3.1.9).

### Results

The MAC 16 tumour produces extensive host body weight loss. In males almost 20% of the total body weight is lost within 30 days after tumour transplantation (Figure 1) and in females as much as 33% when compared with age matched controls. Weight loss occurs even with a small tumour burden and increases with increasing weight of tumour (Figure 2). The first significant loss of weight occurs around 14 days after tumour transplantation when the tumour mass is only ~0.2 g (<1% of the host weight). The host weight reduction occurs without a measurable drop in food intake. Concomitant with the reduction in host weight there is also a decrease in the level of

**Figure 1** Mean body weight of male NMR1 mice either implanted with MAC 16 tumour on day 0 (○) or non tumour-bearing (●).

**Figure 2** Relationship between carcass weight and tumour weight in male NMR1 mice bearing the MAC 16 tumour.

**Figure 3** Blood glucose concentration in tumour-bearing (▲) and non tumour-bearing (●) NMR1 mice after tumour transplantation.

blood glucose in tumour-bearing mice with increasing time after tumour transplantation, which is significantly lower ($P<0.01$) than non-tumour-bearing mice 21 days after tumour transplantation (Figure 3).

The rates of glucose and palmitate oxidation and $CO_2$ and lactate production by a range of human and murine cell lines *in vitro* is shown in Table I. Of the cell lines examined only the MAC 16 is known to produce cachexia in the host. For all cell lines oxidation of glucose *in vitro* exceeds that of palmitate by a factor of $\sim 10^3$. Of the cell lines the MAC 16 shows the highest conversion of both glucose and palmitate to $CO_2$ and the highest conversion of glucose to lactate, even though it has

**Table I** Substrate utilization and product formation by cell lines *in vitro*[a].

| *Cell line* | *Glucose consumed* $\mu mol\,h^{-1}\,10^{-6}$ *cells* | *Lactate production* $\mu mol\,h^{-1}\,10^{-6}$ *cells* | *Glucose lactate (%)* | *Glucose* $CO_2$ $nmol\,h^{-1}\,10^{-6}$ *cells* | *Palmitate* $CO_2$ $nmol\,h^{-1}\,10^{-6}$ *cells* | *Lactate* $CO_2$ | *Doubling time (h)* |
|---|---|---|---|---|---|---|---|
| MAC 16 | 2.6±0.25 | 4.0±0.4 | 80 | 26±4 | 199±16 | 373 | 64 |
| L132 | 1.9±0.2 | 2.5±0.4 | 66 | 13±1.5 | 199±6 | 195 | 48 |
| MAC 15A | 1.5±0.2 | 0.52±0.1 | 17 | 4±0.4 | — | 130 | 19.5 |
| MAC 13 | 0.97±0.06 | 1.86±0.3 | 96 | 12±0.4 | — | 16 | 20 |
| Raji | 0.53±0.02 | 0.54±0.2 | 51 | 10±0.2 | 11±0.2 | 54 | 16 |
| GM 892A | 0.49±0.11 | 0.6±0.2 | 61 | 8±0.4 | 26±0.3 | 76 | 14 |
| TLX5 | 0.49±0.02 | 0.62±0.2 | 63 | 5.3±0.4 | 72±5 | 155 | 14 |
| L1210 | 0.35±0.02 | 0.48±0.2 | 68 | 2.5±0.3 | 16±2 | 212 | 12 |
| K562 | 0.30±0.003 | 0.3±0.1 | 50 | 8.6±1 | 6±0.1 | 35 | 13 |
| GMO621 | 0.27±0.02 | 0.24±0.1 | 44 | 2.9±1 | 26±3 | 83 | 24 |
| HL60 | 0.19±0.02 | 0.16±0.02 | 42 | 6.2±0.2 | 29±5 | 26 | 25 |

[a]Results are averages of 4 determinations carried out in duplicate and are expressed as mean±s.e.

the slowest doubling time of any of the cell lines. These results are similar when expressed either in terms of substrate utilization/production per cell or per milligram of total cellular protein. The ratio of the anerobic to aerobic utilization of glucose is high for TLX5, L1210, L132, MAC 15A and MAC 16. Thus it appears that the substrate requirements for energy production by the MAC 16 tumour are high when compared with other cell lines.

The ability of the MAC 16 tumour to utilize 3-hydroxybutyrate as an energy source depends upon the presence of three enzymes – 3-hydroxybutyrate dehydrogenase, 3 oxo acid CoA transferase and acetoacetyl CoA thiolase. The level of these enzymes in the MAC 16 tumour and in host tissue is shown in Table II. There is no statistical difference in the activity of any of these enzymes from tissues of tumour-bearing and non tumour-bearing mice. The tissue distribution of these enzymes is similar to that previously reported (Tisdale & Brennan, 1983). The activity of 3-hydroxybutyrate dehydrogenase in all tissues is high enough to allow for the utilization of 3-hydroxybutyrate by metabolic oxidation. When compared with normal colon the activity is higher in the MAC 16 tumour suggesting no limitation on the conversion of 3-hydroxybutyrate to aceto-acetate.

Amongst the non-involved mouse tissues the activity of 3 oxo acid CoA transferase is highest in the heart and lowest in the liver as previously reported (Fenselau & Wallis, 1974; Tisdale & Brennan, 1983). The activity of this enzyme is lower in the MAC 16 tumour than any other tissue, except liver, and is significantly lower than that of normal colon ($P<0.002$). Similar specific activities were obtained with the *in vitro* model. The lowest enzyme levels were found in the liver, an organ not regarded as utilizing ketone bodies as metabolic substrates. This result suggests that the MAC 16 tumour may have a limited capacity to metabolize acetoacetate.

High levels of acetoacetyl CoA thiolase are found in kidney, heart and liver. The lowest level is found in the MAC 16 tumour and this activity is significantly ($P<0.003$) lower than normal colon. The high level of this enzyme in liver probably reflects the role of the thiolase in processes other than ketone body utilization.

### Discussion

Tumour cells exhibit considerable flexibility in their ability to utilize energy producing substrates *in vitro*. However, a number of rapidly growing tumours exhibit high rates of aerobic glycolysis and lactate production. The AS-30D rat hepatoma cell line when supplied *in vitro* with glucose as the only energy source derived about 60% of the total cell ATP from glycolysis and 40% from oxidative phosphorylation (Nakashima *et al.*, 1984). Some tumours are unable to utilize fat as a metabolic substrate due to enzyme deficiency. Thus the poorly differentiated hepatoma 7777 exhibited low levels of fatty acyl CoA, no appreciable activity of carnitine palmitoyl transferase and fortified homogenates of the tumour were unable to oxidize palmitate (Fields *et al.*, 1981). In general those tumours which grow slowly and which lack the capacity for glucose phosphorylation readily oxidize fatty acids, while the high glycolysing tumours fail to oxidize fatty acids (Bloch-Frankenthal *et al.*, 1965). Zielke *et al.* (1984) have also shown that oxidation of fatty acids or ketone bodies does not contribute significantly to the energy needs of cultured mammalian cells, and that the apparent requirement for glucose is related to its role in anabolic reactions. In the

**Table II** Activities of enzymes of ketone body metabolism in tissues of NMR 1 mice and in the MAC 16 tumour.

| | *Enzyme activity* ($\mu$ *mol min*$^{-1}$ *mg*$^{-1}$ *protein*) (*Mean* ± *s.e.*) | | |
|---|---|---|---|
| *Tissue* | *3-Hydroxybutyrate dehydrogenase* | *3 oxo acid CoA transferase* | *Acetoacetyl CoA thiolase* |
| Heart | 1.1±0.5 | 14.2±2.21 | 45.7±1.3 |
| Liver | 2.9±0.6 | 0.3±0.17 | 60.0±13.5 |
| Kidney | 2.1±0.6 | 8.5±2.6 | 61.5±6.1 |
| Brain | 6.6±2.3 | 3.1±0.5 | 15.2±4.6 |
| Colon | 2.8±0.2 | 4.4±0.7 | 42.2±4.5 |
| MAC 16 | 5.1±1.2[a] | 1.7±0.5[a] | 16.4±2.2[a] |

[a]$P<0.01$ from normal colon.

present study the rate of palmitate oxidation to $CO_2$ was only about one tenth the rate of $CO_2$ production from glucose even in the MAC 16. This low utilization of fatty acids may explain the reduction in tumour size when the host is fed a high fat diet. Isocaloric consumption of a diet high in fat and protein and low in carbohydrate has been shown to significantly prolong the survival of MCA-sarcoma bearing rats (Demetrakopoulos & Rosenthal, 1982) and a diet high in long chain triglycerides reduced the number of B16 melanoma deposits in the lungs of C57BL/6 mice by two thirds (Magee *et al.*, 1979). Also starvation-induced hypoglycaemia and streptozotocin-induced diabetes has been shown to suppress the growth of Ehrlich ascites tumour in mice (Fung *et al.*, 1985). In the cell lines under study the ratio of lactate to $CO_2$ production from glucose ranged from over 200 to 26. Thus, although the cultures were well aerated production of lactate from glucose appears to be the preferred metabolic reaction.

*In vivo* tumour architecture may play a role in the choice of metabolic substrates. Large solid tumours tend to be poorly vascularized on the inside and a large fraction is consequently hypoxic. Under conditions of low oxygen tension the mitochondiral oxidation pathway may be non-functional and glucose may be the only utilizable metabolic substrate, since the Embden Meyerhoff pathway is the only means of ATP production that does not require oxygen. Indeed, increased Cori cycle activity has been observed in cancer patients with progressive weight loss, showing that lactate production rates are higher in these patients (Holroyde *et al.*, 1975). If the glucose formed from lactate in the liver is utilized by the tumour to produce more lactate, then this represents an energy burden on the host, which has been considered responsible for the tumour-induced weight loss (Gold, 1976).

The high glucose consumption by the MAC 16 tumour *in vitro* may be responsible for the hypoglycaemia in the host if the same situation occurs *in vivo*, although substrate utilization *in vitro* may have no bearing whatsoever to the situation *in vivo*. A tumour mass of 1 g of viable cells would consume about 11 g of glucose per day, which represents a considerable burden on the host. If the glucose was not derived from external sources and was obtained by gluconeogenesis from lactate the host would need to consume an extra 3.4 g of fat, or derive it from its adipose supply, in order to supply sufficient ATP to drive the Cori cycle. Even a small tumour burden would impose a considerable strain on the host, which may account for the rapid depletion of fat deposits in mice bearing the MAC 16 tumour.

We and others (Tisdale & Brennan, 1983; Magee *et al.*, 1979; Williams & Matthaei, 1981) have considered the possibility of reversing the cachectic effect of certain tumours by the induction of systemic ketosis. Metabolism of ketone bodies to produce ATP requires oxygen and thus might be expected to be low in the anoxic tumour fraction. In addition the extent to which 3-hydroxybutyrate serves as a respiratory fuel is thought to be governed by the level of 3 oxo acid CoA transferase (Williamson *et al.*, 1971), although some tumours have acquired the enzyme acetoacetyl CoA synthetase, which enables the direct utilization of acetoacetate (Tisdale, 1984). Levels of 3 oxo acid CoA transferase have been reported to be decreased or absent in a range of murine (Fields *et al.*, 1981; Tisdale & Brennan, 1983) and human tumours (Fredericks & Ramsey, 1978) suggesting an impaired ability to metabolize ketone bodies. The MAC 16 is a well differentiated adenocarcinoma and enzyme activity has been compared with normal colon. On this basis MAC 16 also displays a low activity of 3 oxo acid CoA transferase and might be expected to metabolize 3-hydroxybutyrate at a reduced rate. Indeed under a dietary regime in which a high proportion of the energy is supplied as medium chain triglycerides tumour growth is inhibited *in vivo* without any alteration in the levels of ketone body metabolizing enzymes (Tisdale *et al.*, 1986). These results suggest that it may be possible to differentially feed the host at the expense of the tumour.

This work has been supported by a grant from the Cancer Research Campaign. The authors would like to thank Drs M.C. Bibby and J.A. Double for providing the MAC tumours.

### References

BIBBY, M.C., DOUBLE, J.A., ALI, S.A., FEARON, K.C.H., BRENNAN, R.A. & TISDALE, M.J. (1986). Characterisation of a transplantable adenocarcinoma of the mouse colon producing cachexia in recipient animals. *J. Natl Cancer Inst.* (in press).

BLOCH-FRANKENTHALL, L., LANGAN, J., MORRIS, H.P. & WEINHOUSE, S. (1965). Fatty acid oxidation and ketogenesis in transplantable liver tumors. *Cancer Res.*, **25**, 732.

DEMETRAKOPOULOS, G. & ROSENTHAL, A. (1982). Prolonged survival of MCA-sarcoma bearing rats fed a low carbohydrated diet. *Proc. Am. Assoc. Cancer Res.*, **23**, 10.

DOUBLE, J.A. & BALL, C.R. (1975). Chemotherapy of transplantable adenocarcinoma of the colon in mice. *Cancer Chemother. Rep.*, **59**, 1083.

FENSELAU, A. & WALLIS, K. (1974). Comparative studies of 3-oxo acid coenzyme A transferase from various rat tissues. *Biochem. J.*, **142**, 619.

FIELDS, A.L.A., WOLMAN, S.L., CHEEMA-DHADLI, S., MORRIS, H.P. & HALPERIN, M.L. (1981). Regulation of energy metabolism in Morris hepatoma 7777 and 7800. *Cancer Res.*, **41**, 2762.

FREDERICKS, M. & RAMSEY, B.B. (1978). 3-Oxo acid coenzyme A transferase activity in brain and tumours of the nervous system. *J. Neurochem.*, **31**, 1529.

FUNG, K.P., CHAN, T.W. & CHOY, Y.M. (1985). Suppression of Ehrlich ascites tumor growth in mice by starvation and streptozotocin-induced diabetes. *Cancer Lett.*, **28**, 273.

GOLD, J. (1976). Hydrazine sulphate in the treatment of cancer. *Cancer Treat. Rep.*, **60**, 964.

GUTMAN, I. & WAHLEFIELD, A.N. (1974). L-(+)-Lactate determination with lactate dehydrogenase and NAD. In *Methods of Enzymatic Analysis*, Bergmeyer, H.U. (ed) **4**, p. 1464. Academic Press: London and New York.

HOLROYDE, C.P., GABUZDA, T.G., PUTNAM, R.C., PONUL, A. & REICHARD, G.A. (1975). Altered glucose metabolism in metastatic carcinoma. *Cancer Res.*, **35**, 3710.

LOWRY, O.H., ROSEBROUGH, N.J., FARR, A.L. & RANDALL, R.J. (1951). Protein measurement with the folin phenol reagent. *J. Biol. Chem.*, **193**, 265.

MAGEE, B.A., POTEZNY, N., ROFE, A.M. & CONYERS, R.A.J. (1979). The inhibition of malignant cell growth by ketone bodies. *Aust. J. Exp. Biol. Med. Sci.*, **57**, 529.

NAKASHIMA, R.A., PAGGI, M.G. & PEDERSEN, P.L. (1984). Contributions of glycolysis and oxidative phosphorylation to adenosine 5′-triphosphate production in A5-30D hepatoma cells. *Cancer Res.*, **44**, 5702.

TISDALE, M.J. (1984). Role of acetoacetyl-CoA synthetase in acetoacetate utilization by tumor cells. *Cancer Biochem. Biophys.*, **7**, 101.

TISDALE, M.J. & BRENNAN, R.A. (1983). Loss of acetoacetate coenzyme A transferase activity in tumours of peripheral tissues. *Br. J. Cancer*, **47**, 293.

TISDALE, M.J., BRENNAN, R.A. & FEARON, K.C.H. (1985). Enzymatic and metabolic profiles in a cachexia model. *Br. J. Cancer*, **51**, 593.

TISDALE, M.J., BRENNAN, R.A. & FEARON, K.C.H. (1986). Reduction of weight loss and tumor size in a cachexia model by a high fat diet. *J. Natl Cancer Inst.* (submitted).

WILLIAMS, J.F. & MATTHAEI, K.I. (1981). Cancer induced by body wasting. A review of cancer cachexia and a hypothesis concerning the molecular basis of the condition. *J. Clin. Sci. ASEAN*, **2**, 158.

WILLIAMSON, D.H., BATES, M.W., PAGE, M.A. & KREBS, H.A. (1971). Activities of enzymes involved in acetoacetate utilization in adult mammalian tissues. *Biochem. J.*, **121**, 41.

ZIELKE, H.R., ZIELKE, C.L. & OZAND, P.T. (1984). Glutamine: a major energy source for cultured mammalian cells. *Fed. Proc.*, **43**, 121.